# Changes in Quinoa Seed Fatty Acid Profile Under Heat Stress Field Conditions

**DOI:** 10.3389/fnut.2022.820010

**Published:** 2022-03-28

**Authors:** Javier Matías, María José Rodríguez, Sara Granado-Rodríguez, Verónica Cruz, Patricia Calvo, María Reguera

**Affiliations:** ^1^Agrarian Research Institute “La Orden-Valdesequera” of Extremadura, Centro de Investigaciones Científicas y Tecnológicas de Extremadura, Badajoz, Spain; ^2^Technological Institute of Food and Agriculture of Extremadura, Centro de Investigaciones Científicas y Tecnológicas de Extremadura, Badajoz, Spain; ^3^Department of Biology, Universidad Autónoma de Madrid, Madrid, Spain

**Keywords:** quinoa, heat stress, fatty acids, seed oil content, seed nutritional quality, erucic acid, ω6/ω3 ratio

## Abstract

The nutritional quality of quinoa is often related to the high protein content of their seeds. However, and despite not being an oilseed crop, the oil composition of quinoa seeds is remarkable due to its profile, which shows a high proportion of polyunsaturated fatty acids (PUFAs), particularly in essential fatty acids such as linoleic (ω-6) and α-linolenic (ω-3). In line with this, this study aimed at evaluating the effect of elevated temperatures on the oil composition of different quinoa cultivars grown in the field in two consecutive years (i.e., 2017 and 2018). In 2017, heat stress episodes resulted in a reduced oil content and lower quality linked to decreased ratios of oleic acid:linoleic acid, larger omega-6 (ω-6) to omega-3 (ω-3) ratios, and lower monounsaturated fatty acid (MUFA) and higher PUFA contents. Furthermore, the correlations found between mineral nutrients such as phosphorous (P) and the contents of oleic and linoleic acids emphasize the possibility of optimizing oil quality by controlling fertilization. Overall, the results presented in this study show how the environmental and genetic factors and their interaction may impact oil quality in quinoa seeds.

## Introduction

The demand for novel healthy foods and novel food ingredients is continuously growing parallel to the expansion of their market (1). In line with this, quinoa (*Chenopodium quinoa* Willd.), which is well recognized worldwide due to the high nutritional value of its edible seeds (2–7), is being incorporated into novel food products (8). Quinoa seeds are gluten-free and rich in minerals, vitamins, and dietary fiber, and various bioactive compounds are also present in significant amounts in different parts of the plant. The oil content of quinoa seed ranges from 2 to 10%, with an average of 5.0–7.2% (3), with yields between 80 and 400 kg of oil ha^–1^, which reveal quinoa as a new potential oilseed crop (9), although those values are lower than those found in common oilseed crops, such as sunflower (40–50%), rapeseed (30–50%), or soybean (20%) (10). Furthermore, the nutritional quality of quinoa oil is remarkable (3, 4, 11, 12). It is rich in polyunsaturated fatty acids (PUFAs), particularly in essential fatty acids as linoleic (ω-6) and α-linolenic (ω-3), which have healthy properties with beneficial effects on cardiovascular risk, improving insulin sensitivity (12). Moreover, the ω-6 to ω-3 ratio (ω-6/ω-3) of quinoa is close to the recommended daily intake in a healthy diet (5:1–10:1) (6, 13). Normally, western diets are associated with deficient levels of ω-3 and excessive amounts of ω-6, resulting in a ω-6 to ω-3 ratio higher than 15:1, which in turn results in health risks associated with different disorders that may be promoted, including cardiovascular diseases (14, 15).

Essential fatty acids, such as linoleic acid (18:2, n-6) or alpha-linolenic acid (18:3, n-3) fatty acids, cannot be synthesized by the human body and need to be obtained from food (15, 16). The amount of saturated fatty acids (SFAs) and unsaturated fatty acids varies in different oil crops (15). Also, fatty acid composition depends on the environmental conditions (17–19). It should be noted that the oil content has an important role in grain or seed storage since it can be degraded, resulting in oxidative rancidity and altering its nutritional and organoleptic quality and its germination power (20, 21). For instance, higher PUFA levels can lead to oil oxidation, reducing the oil quality (22). In contrast, lipid degradation into glycerol and free fatty acids (lipid hydrolysis) is carried out by a group of enzymes known as lipases, which can be triggered by lipid oxidation (21, 23). Interestingly, the growing embryo has been reported to show the largest lipase activity (20), and overall, both lipid oxidation and degradation increase during long-term seed dry storage, affecting seed germination and viability.

Quinoa, an herbaceous plant belonging to the Amaranthaceae family, has acquired a significant interest beyond its center of origin, the Andean region (24). Quinoa has a great capacity for adaptation to a wide range of environmental conditions linked to its huge genetic diversity (11, 25). Consequently, an increasing interest in quinoa cultivation has been experienced in the past decades in different geographical areas, resulting in the global expansion of this crop (26). Particularly in Europe, there is a great interest in growing quinoa (27). In fact, it was introduced in northern European countries (i.e., England, Denmark, and Netherlands) in the late 1970s and early 1980s, and nowadays, it is also cultivated in the Mediterranean region (28). In Spain, quinoa has spread significantly in recent years, particularly in the southern part of the country (29).

Quinoa is a short-day plant, being sensitive to photoperiod at all stages of development, particularly at the reproductive stage (30, 31). This has been pointed out as one of the main obstacles for introducing quinoa in Europe (32). Accordingly, new varieties that are less sensitive to photoperiod and better adapted to the European conditions have been developed (26). However, these newly bred varieties are not always well adapted to the elevated temperatures that are frequent in the Mediterranean areas, resulting in important yield penalties. In this regard, variations in the chemical composition of quinoa seeds have also been reported under heat stress field conditions (29, 33). These include changes in the fatty acid profile of quinoa, which was shown to be affected by the environmental conditions (9).

Lipids play an important role in the responses to environmental stresses, being the major compounds of biological membranes. The change in lipid metabolism induced by heat stress-induced changes in lipid metabolic of plants has been studied in species such as oilseeds (sunflower (34), rapeseed (35), or cereals (wheat (*Triticum aestivum* L.), maize (*Zea mays* L.), or sorghum [*Sorghum bicolor* (L.) Moench], observing, generally, a replacement of the highly unsaturated lipids by less unsaturated ones (36). The oil quality, including its nutritional value, flavor, and physical properties such as oxidative stability, depends on the composition and distribution of the fatty acids (37). Furthermore, in contrast to drought and salinity, the heat stress response in quinoa has been poorly studied (38, 39). The specific influence of heat stress on the fatty acid profile of quinoa seeds is still missing (40). Considering the current climate change scenario, which includes more recurrent episodes of elevated temperatures (41) that may cause detrimental impacts on agriculture, food safety, and security (42, 43), it is crucial to evaluate the effects of heat stress episodes on seed yield and quality in food crops. Therefore, in this study, aiming at further exploring this aspect, we evaluated five quinoa varieties cultivated under field conditions in Southwestern Europe, where episodes of elevated temperatures are quite frequent, aiming to shed light on changes in the fatty acid profile of quinoa oil under heat stress conditions.

## Materials and Methods

### Location, Experimental Design, and Crop Management

A 2-year field experiment was conducted in two consecutive years (2017 and 2018) at an experimental farm that belongs to the Center for *Scientific and Technological Research of Extremadura* (CICYTEX), located in Southwest Spain (latitude 38° 51′10″ N; longitude 6° 39′10″ W). Experimental design, management, climate, and soil characteristics of the experimental site were described in a previous study (33).

### Analysis and Measurements

Protein, mineral, and fiber contents were determined as described in previous studies (29, 33). Fat content was analyzed according to AOAC Official Methods 930.09 (44). Fatty acid methyl esters (FAMEs) from the oil samples were obtained by alkaline treatment using 2N KOH in methanol at room temperature; FAME separation and quantification gas chromatography (GC) were performed according to the European Commission Regulation (EEC) No 2568/91 (45) using an Agilent 6890A Gas Chromatograph (Agilent Technologies, CA, United States) equipped with a flame ionization detector (FID) and column Supelco DB-23 60 m × 0.25 mm × 0.25 mm (Agilent Technologies). The injector and detector temperatures were 260 and 280°C, respectively. The column was maintained at 185°C for 4 min, followed by a heating rate of 5°C/min to a temperature of 220°C, which was maintained for 9 min, and then raised again from 185°C. The ultra-pure gas flows were of 1.2 ml/min (carrier gas—helium 5.6), 25 ml/min [make-up gas nitrogen (N)], 400 ml/min (synthetic air), and 40 ml/min (hydrogen flame gas), with a split injection ratio of 1/100. The identification has been carried out by comparing the peaks with a certified reference material (Supelco 37 Component FAME Mix, 1 × 1 ml, varied concentration in dichloromethane) produced in a laboratory accredited by ISO/IEC 17025 and ISO Guide 34. The results are expressed in relative percentage of each fatty acid.

Aiming at confirming the FAME peaks identified and quantified by GC, a gas chromatography-mass spectrometer (GC-MS) analysis was performed. The system was equipped with a 453-GC and a Scion triple quadrupole mass detector (Bruker Daltonics, Leipzig, Germany), using the same GC column described above. Helium was used as carrier gas at a flow rate of 1 ml/min. The injection volume was 1 ml, and the split ratio was 50. The inlet temperature was 250°C. The temperature of the transfer interface and ion source was 250 and 280°C, respectively. The temperature program was set as follows: the column was maintained at 150°C for 2 min, followed by a heating rate of 3°C/min to 230°C, which was maintained for 5 min and followed by a heating rate of 5°C/min to 250°C, and then raised again from 150°C. For qualitative analysis, the full scan mode was used to collect the total ion current chromatogram (TIC) and the mass spectrum of the analytes. The selective ion monitoring mode (SIM) was employed for confirmation analysis, and the FAMEs were identified by comparing the retention time with those of derivatized standards and by comparison of mass spectra with the NIST/EPA/NIH mass spectrum library.

### Statistical Analysis

For those variables where normality and equal variances could be assumed, a one-way ANOVA test was performed, followed by a Tukey’s *post hoc* test, to perform multiple comparisons at a probability level of 5% (*p* < 0.05). A Kruskal–Wallis test by ranks was performed when data did not present a normal distribution, and a Welch’s ANOVA test followed by a Games–Howell *post hoc* test was performed when variances were not equal, both at a probability level of 5% (*p* < 0.05). The year and variety were treated as fixed factors. Normality and equality of variances of the data were determined using Shapiro–Wilk and Levene’s tests, respectively. A Pearson’s correlation coefficient was used to evaluate the statistical association between variables. The SPSS Statistics 22.0 (SPSS Inc., Chicago, United States) package was used for the statistical analyses performed in this study.

## Results

All varieties were able to complete their life cycle in both years (2017 and 2018). Generally, yields in 2018 were significantly higher compared with 2017. The agronomic performance and the results of nutritional and mineral composition of seeds, together with the meteorological conditions of 2017 and 2018, are reported in a previous study (33).

### Seed Oil Composition

**FIGURE 1:**
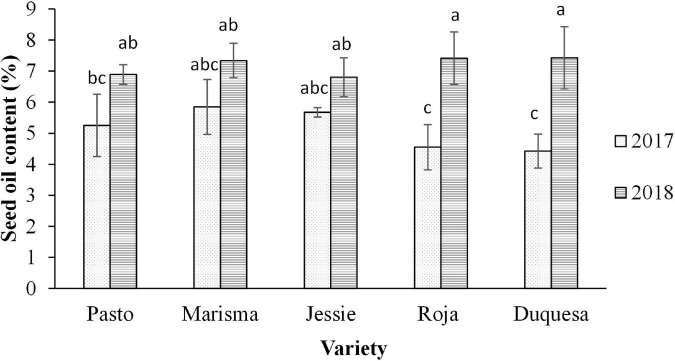
Seed oil content of five quinoa varieties harvested in 2 years (2017 and 2018). Total seed oil content is expressed as percentage (%). Error bars correspond to the SDs. Bars that do not share the same letters show statistically significant differences following an ANOVA test and Tukey’s *post hoc* test at a *p*-value < 0.05.

Oil content was determined in seeds harvested in 2017 and 2018 (Figure 1). The average oil content was significantly higher in 2018 (7.2%) compared with 2017 (5.1%). However, differences between years only appeared in medium to long-duration varieties (Roja and Duquesa), with a lower average content in 2017 compared with 2018 in both varieties (with approximately 4.5% total oil content).

**FIGURE 2:**
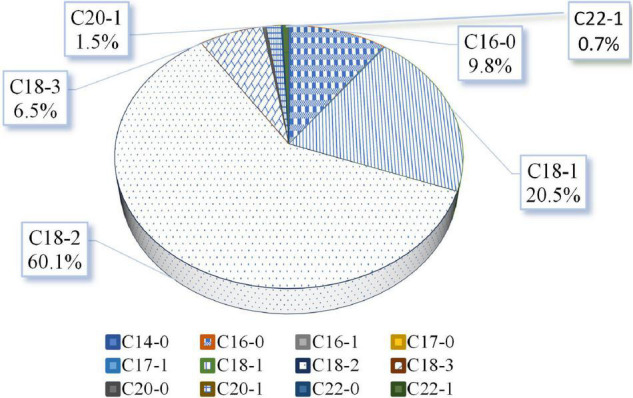
Fatty acid composition of seeds harvested from five quinoa varieties grown in 2 years (2017 and 2018) on field experiments. Mean values are shown for each fatty acid quantified, and the values are presented as percentage (%). The fatty acids detected included the following: myristic—C14:0, palmitic—C16:0, palmitoleic—C16:1, margaric—C17:0, margaroleic—C17:1, stearic—C18:0, oleic—C18:1, linoleic acid—C18:2, α-linolenic—C18:3, gadoleic—C20:1, behenic—C20:1, and erucic—C22:1.

As observed in Figure 2, the most abundant fatty acid was linoleic acid (C18:2), which reached 60.1% of the total seed oil content. This was followed by oleic acid (C18:1) (20.5%) and palmitic acid (C16:0) (9.8%).

**FIGURE 3:**
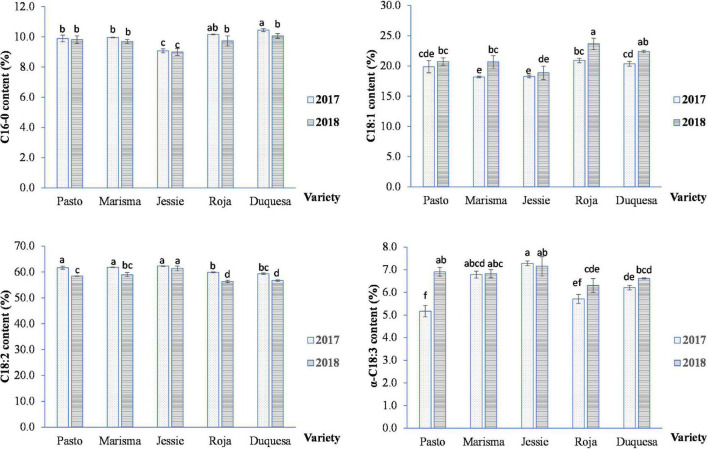
Major fatty acid contents of quinoa seeds obtained from five varieties and 2 cultivation years (2017 and 2018). Error bars correspond to the SDs. Bars that do not share the same letters show statistically significant differences following an ANOVA test and Tukey’s *post hoc* test (C16:0, C18:1, C18:2, and α-C18:3) at a *p*-value < 0.05.

The major fatty acid composition varied with the quinoa variety and cultivation year, as shown in Figure 3. The palmitic acid (C16:0) mean value in 2017 (9.9%) was higher than in 2018 (9.7%). The most remarkable difference in palmitic acid content appeared in medium-long life cycle varieties, especially in Duquesa, which showed significant differences between years. Jessie showed the lowest palmitic acid content. The results indicated that the fatty acid composition of the short life cycle variety Jessie was different compared with the medium-long life cycle varieties (Roja and Duquesa). The oleic acid content (C18:1) was significantly higher in 2018 appearing with differences in Marisma and in medium-long life cycle varieties (Roja and Duquesa), and these last varieties showed the highest C18:1 content and Jessie the lowest one. On the contrary, the linoleic acid (C18:2) content was, on average, higher in 2017 except for Jessie, which did not show significant differences. Roja and Duquesa achieved a lower content in both years. Regarding the linolenic acid content, 2018 showed higher contents of α-linolenic (6.8%) compared with 2017 (6.2%). In Pasto and medium-long life cycle varieties (Roja and Duquesa), the α-linolenic content varied between years, while in Jessie and Marisma, the content was similar.

**FIGURE 4:**
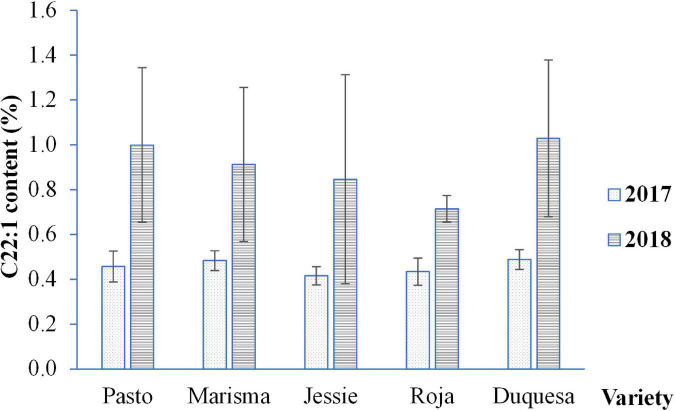
Erucic acid (C22:1) content in quinoa seeds obtained from five varieties and 2 cultivation years (2017 and 2018). Error bars correspond to the SDs. Bars that do not share the same letters show statistically significant differences following a Welch’s ANOVA test followed by a Games–Howell *post hoc* test at a *p*-value < 0.05.

As shown in Table 1, different fatty acids were present in lower amounts (minor fatty acids). Generally, the cultivation year did not significantly affect the composition except for C18:0, C20:1, and C22:0. Among the minor fatty acids, gadoleic acid (C20:1) was the most abundant one with a total average content of 1.50%, being Jessie and Marisma the varieties that presented the lowest amounts for this fatty acid (1.43%) and Pasto, the highest (1.55%). When analyzing changes between years, it was observed that differences only appeared in Roja, whose gadoleic acid content in 2018 (1.58%) was higher than in 2017 (0.49%). Pentadecanoic acid (C15:0), palmitoleic acid (C16:1), and margaric acid (C17:0) were detected in very low amounts (<0.1%), especially the palmitoleic acid that was not detected in 2017. Average stearic acid (C18:0) content was 0.46%, showing similar values between years except for Roja that presented a higher amount in 2018. The average content of margaroleic acid was 0.10%, being higher in 2017 in all varieties with the exception of Jessie, in which the margaroleic acid content was the same in both years. The arachidic acid achieved an average value of 0.35%, with small differences between years in all varieties, except for Duquesa, in which a significantly higher content was achieved in 2017. The behenic acid content ranged from 0.05 to 0.19%, being significantly higher in 2018 (0.17%) than in 2017 (0.12%). The lowest contents were found in Jessie for both years. Interestingly, among the fatty acids detected, erucic acid was present in the analyzed quinoa seeds (Figure 4). No differences were found among varieties, although the variability in the erucic acid contents increased in 2018 for most of the varieties except for Roja (as reflected by the error bars).

**TABLE 1:** Minor fatty acid contents in quinoa seeds harvested from five different varieties cultivated in 2 years (2017 and 2018).

Fatty acid (Relative%)	Pasto		Marisma		Jessie		Roja		Duquesa	
	2017	2018	2017	2018	2017	2018	2017	2018	2017	2018
C14:0	0.23 ± 0.01^bc^	0.24 ± 0.03^b^	0.31 ± 0.01^a^	0.22 ± 0.03^bc^	0.17 ± 0.02^cd^	0.14 ± 0.03^d^	0.22 ± 0.01^bc^	0.19 ± 0.02 ^bcd^	0.17 ± 0.01^bcd^	0.14 ± 0.01^d^
C15:0	0.07 ± 0.01^ ab^	0.06 ± 0.01^ab^	0.07 ± 0.01^ab^	0.01 ± 0.01^b^	0.08 ± 0.01^a^	0.07 ± 0.01^ab^	0.07 ± 0.01^ab^	0.06 ± 0.01^ab^	0.07 ± 0.01^ab^	0.08 ± 0.01^a^
C16:1	0.00 ± 0.00	0.02 ± 0.01	0.00 ± 0.00	0.01 ± 0.01	0.00 ± 0.00	0.06 ± 0.01	0.00 ± 0.00	0.02 ± 0.02	0.00 ± 0.00	0.01 ± 0.02
C17:0	0.05 ± 0.01^abc^	0.03 ± 0.01^b^	0.05 ± 0.01^abc^	0.01 ± 0.01^abc^	0.06 ± 0.01^ac^	0.02 ± 0.01^bc^	0.05 ± 0.01^a^	0.02 ± 0.01^ab^	0.07 ± 0.02^abc^	0.02 ± 0.01^abc^
C17:1	0.14 ± 0.06	0.09 ± 0.04	0.08 ± 0.03	0.07 ± 0.01	0.04 ± 0.01	0.04 ± 0.01	0.14 ± 0.01	0.10 ± 0.01	0.16 ± 0.04	0.13 ± 0.03
C18:0	0.42 ± 0.03^cd^	0.44 ± 0.03^cd^	0.39 ± 0.02^d^	0.46 ± 0.05^bcd^	0.43 ± 0.02^cd^	0.49 ± 0.04^abc^	0.42 ± 0.02^cd^	0.56 ± 0.05^a^	0.49 ± 0.02^abc^	0.54 ± 0.03^ab^
C20:0	0.34 ± 0.01^bc^	0.34 ± 0.02^bc^	0.33 ± 0.01^bc^	0.31 ± 0.02^c^	0.34 ± 0.02^bc^	0.31 ± 0.04^ab^	0.37 ± 0.03^abc^	0.38 ± 0.02^ab^	0.43 ± 0.03^a^	0.36 ± 0.03^bc^
C20:1	1.51 ± 0.13^abcd^	1.59 ± 0.07^abc^	1.33 ± 0.03^bcd^	1.55 ± 0.07^d^	1.40 ± 0.01^d^	1.47 ± 0.04^d^	1.42 ± 0.08^ab^	1.66 ± 0.05^abc^	1.49 ± 0.06^a^	1.58 ± 0.07^bcd^
C22:0	0.13 ± 0.03^ab^	0.19 ± 0.02^a^	0.11 ± 0.01^bc^	0.17 ± 0.02^ab^	0.05 ± 0.01^c^	0.12 ± 0.03^b^	0.14 ± 0.01^ab^	0.17 ± 0.02^ab^	0.16 ± 0.01^ab^	0.19 ± 0.05^a^

*Myristic acid (C14:0); pentadecanoic acid (C15:0); palmitoleic acid (C16:1); margaric acid (C17:0); margaroleic acid (C17:1); stearic acid (C18:0); arachidic acid (C20:0); gadoleic acid (C20:1); and behenic acid (C22:0). Different superscript letters in the same row indicate significant differences following an ANOVA test and Tukey’s post hoc test (C14:0, C15:0, C18:0, C20:0, C20:1, and C22:0) and a Welch’s ANOVA test followed by a Games-Howell post hoc test (C17:0 and C17:1) and Kruskal–Wallis test (C16:1) at a p-value < 0.05. The results are expressed in relative percentage.*

**FIGURE 5:**
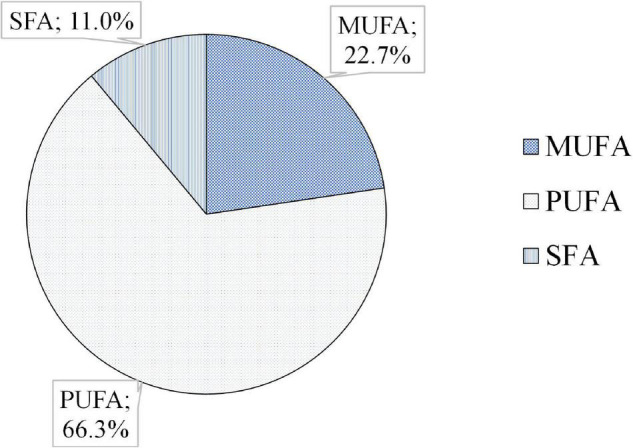
Average content of polyunsaturated fatty acids (PUFA), monounsaturated fatty acids (MUFAs), and saturated fatty acids (SFA) in seeds of five quinoa varieties harvested in 2 years (2017 and 2018).

A simultaneous analysis of saturated and unsaturated was performed (Figure 5). The PUFAs were the most abundant ones (66.3%), followed by the monounsaturated fatty acids (MUFAs) (22.7%), and finally, the SFAs (11.0%).

**FIGURE 6:**
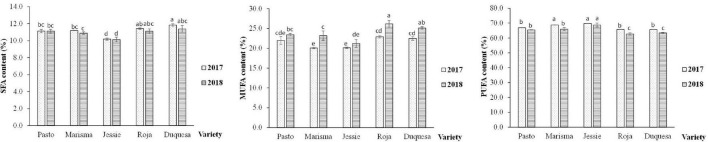
Polyunsaturated fatty acid, MUFA, and SFA contents in quinoa seeds obtained from five varieties and 2 cultivation years (2017 and 2018). Vertical bars mean SD. Bars that do not share the same letters show statistically significant differences following an ANOVA test and Tukey’s *post hoc* test (SFA) and a Welch’s ANOVA test followed by a Games-Howell *post hoc* test (MUFA, PUFA) at a *p*-value < 0.05. SFA: C14:0 + C15:0 + C16:0 + C17:0 + C18:0 + C20:0 + C22:0. MUFA: C16:1 + C17:1 + C18:1 + C20:1 + C22:1. PUFA: C18:2 + C18:3.

**FIGURE 7:**
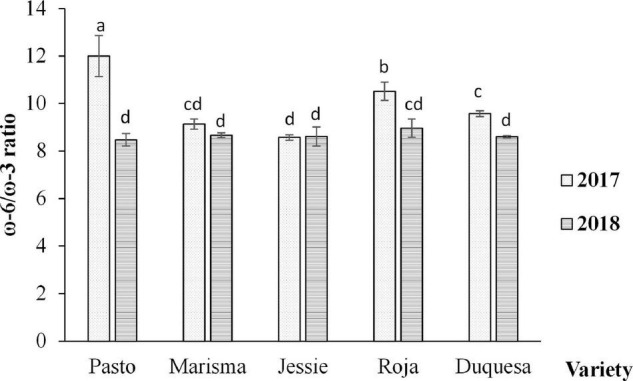
Ratio of omega-6 (ω-6) to omega-3 (ω-3) essential fatty acid (EFA) contents in quinoa seeds obtained from five varieties and 2 cultivation years (2017 and 2018). Error bars correspond to the SDs. Bars that do not share the same letters show statistically significant differences following an ANOVA test and Tukey’s *post hoc* test at a *p*-value < 0.05. Total (ω-3): α-linolenic—C18:3. Total (ω-6): linoleic acid—C18:2.

Nonetheless, the fatty acid distribution, according to the saturation degree and the ratio of omega-6 (ω-6) to omega-3 (ω-3) fatty acids, changed with the variety and the cultivation year, as shown in Figures 6, 7, respectively.

The content of SFA did not vary significantly between years among varieties. However, significant differences were found in MUFA and PUFA contents in Marisma and in medium-long life cycle varieties (Roja and Duquesa), achieving higher values of MUFA and lower levels of PUFA in 2018 (Figure 6). The highest contents of SFA and MUFA were achieved by the varieties with medium-long life cycles (Roja and Duquesa), while the lowest content appeared in the short life cycle variety (Jessie). On the contrary, in both years, the PUFA content was significantly higher in the short cycle variety (Jessie) compared with the medium-long cycle varieties (Roja and Duquesa). Furthermore, the ratio of ω-6 to ω-3 varied depending on the variety and the year (Figure 7). The lowest ratio (8.6, on average) was detected in Jessie in both years. The ratio of ω-6 to ω-3 was higher in 2017 than in 2018, except for Jessie, in which similar values were observed. The highest ratio was achieved by Pasto in 2017 (12.0), followed by Roja (10.5) and Duquesa (9.6).

**FIGURE 8:**
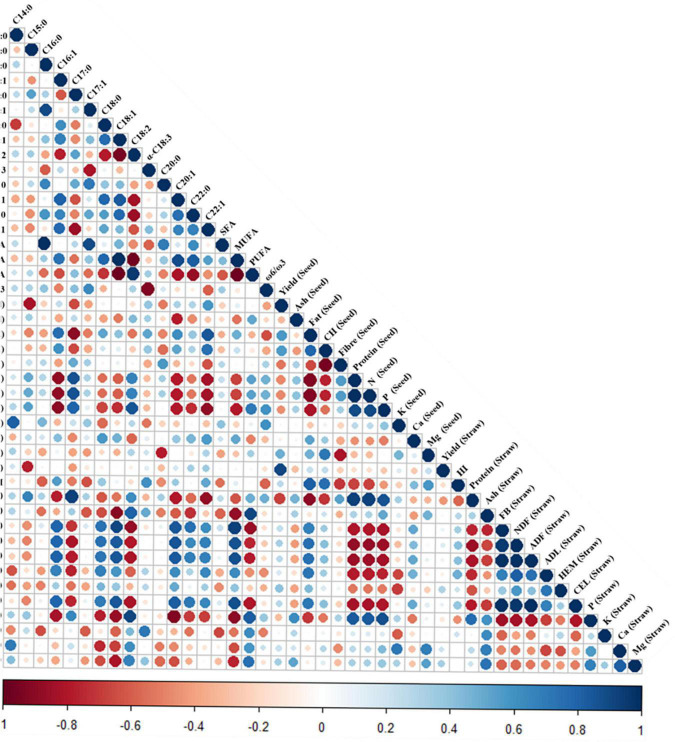
Correlogram of variables measured. Pearson’s correlation coefficients (*r*) are given when the correlation between variables is statistically significant (*p* < 0.05). Red cells indicate negative correlations, and blue cells show positive correlations. The variables considered in the correlogram were as follows: seed fatty acid contents (palmitic—C16:0, stearic—C18:0, oleic—C18:1, linoleic acid—C18:2, α-linolenic—C18:3, and erucic—C22:1), the PUFA, MUFAs, and SFAs, the ratio of omega-6 (ω-6) to omega-3 (ω-3) (ω-6/ω-3), seed yield, seed ash and oil, seed carbohydrates (CH), seed fiber, protein and N, P, K, Ca, and Mg contents, straw yield, harvest index (HI), straw protein, ash, and fiber contents (FB), straw neutral detergent fiber (NDF), acid detergent fiber (ADF), acid detergent lignin (ADL), hemicellulose (HEM), cellulose (CEL), and P, K, Ca, and Mg contents (31).

As observed in Figure 8, different correlations were observed among the analyzed variables. For instance, there was a strong negative correlation between the seed fat content and the seed and straw protein and N contents (−0.9, and −0.9, respectively). Also, seed fat was negatively correlated with the seed and straw phosphorous (P) contents (−0.8 and 0.7, respectively) and positively correlated with the harvest index (HI) (0.81) and the straw Fiber (FB) (0.67), neutral detergent fiber (NDF) (0.76), acid detergent fiber (ADF) (0.74), acid detergent lignin (ADL) (0.72), and cellulose (CEL) (0.71). In contrast, the seed P content positively correlated with the margaric acid (17:0) (0.8), the linoleic acid (C18:2) (0.86) fatty acids, and the seed protein content (0.67) and negatively correlated with the palmitoleic acid (C16:1) (−0.9), gadoleic acid (C20:1) (−0.8), erucic acid (C22:1), and the seed fat contents (−0.8). The oleic acid (C18:1) content, which was the major MUFA found in quinoa seeds (Figure 2), showed a strong negative correlation with the linoleic acid (C18:2) content (−0.9) and also with the PUFA (−1), the straw P (−0.9), and the ash (−0.9) contents and a positive correlation with the MUFA content (0.99) and the straw FB (0.9), NDF (0.83), and ADF (0.84). The linoleic acid (C18:2), being the main PUFA found in quinoa seeds (Figure 2), showed an opposed pattern to the oleic acid (C18:1) with not only a strong positive correlation with the seed P content, as mentioned, but also with the straw ash (0.8), P, and PUFA contents (0.86 and 0.95, respectively), while a negative correlation with the MUFA content (−1), the straw FB (−0.9), NDF (0.8X), ADF (−0.8), and CEL (−0.8). Interestingly, the P straw content showed remarkable negative correlations with the palmitoleic acid (C16:1) (−0.8), the stearic acid (C18:0) (−0.8), the oleic acid (C18:1) (−0.9), the gadoleic acid (C20:1) (−0.9), and the straw FB (−0.8), NDF (−0.8), ADF (−0.8), and CEL (−0.8), and as shown with the seed P content, positive correlations with the seed protein (0.84) and the linoleic acid (C18:2) (0.91) contents. Furthermore, seed and straw proteins showed a similar correlation pattern being positive with the margaric acid (C17:0) (0.87 and 0.93, respectively) and negative with the palmitoleic acid (C16:1) (−0.9 and −0.8, respectively), the gadoleic acid (C:20:1) (−0.8 and −0.8, respectively), and the erucic acid (C22:1) (−0.8 and −0.9, respectively).

## Discussion

Quinoa is considered a highly nutritious food crop due to the compositional characteristics of its seeds (2–7). It not only contains all the essential amino acids, remarkable proteins (15–20%), mineral contents, and fibers (ranging between 13% and 23%, on average) but also is rich in oil (2–10%) (4–6, 46–50). Among the lipid content, the unsaturated fatty acids appeared as the most abundant fatty acids in quinoa seeds (4, 12). As our study revealed, the PUFA) reached almost 70% of the unsaturated fatty acid content supporting the high nutritional quality of its oil being close to previously reported contents in quinoa (51). Nonetheless, other studies have found lower PUFA contents in quinoa (52). The average oil composition of quinoa seeds was mainly composed of linoleic acid (60.1%), oleic acid (20.5%), palmitic acid (9.8%), and α-linolenic acid (6.5%) (Figure 2). Those values are similar to those determined by other authors (7, 9, 27, 47, 53, 54). In this study, the average linolenic acid content was slightly higher than most levels reported by those authors for that fatty acid, while, on the contrary, the oleic acid was slightly higher, resulting in both cases within the ranges of those found by others (40).

The three 18-carbon (C18) compounds 18:1 (oleic), 18:2 (linoleic), and 18:3 (α-linolenic) were the main unsaturated fatty acids, which normally occur in the majority of seeds (55). However, both oil content and composition were affected by the elevated temperatures and radiation during seed filling of 2017 (33). In line with this, it has been previously reported that environmental conditions can affect the nutritional composition of quinoa seeds (56, 57). In this study, we observed that the average oil content decreased almost to 30% under the high temperatures registered in 2017. Furthermore, the heat stress impact on the oil content was higher in medium-long cycle varieties (Roja and Duquesa), with differences up to 40%, which can be explained in those varieties the temperatures during the seed filling were higher. Accordingly, it is well known that high temperatures at the seed-filling stage can affect the content and composition of oil in crops, as observed in sunflower, soybean, or rapeseed (17, 58–60).

According to Canvin (61), soil N availability is augmented with increased temperatures, resulting in larger protein contents and reduced fat contents due to an altered N partitioning among the carbon skeletons. Our results support this hypothesis, as in 2017, the protein content (18.7%) increased 30% compared with 2018 (14.4%) (33) and decreased oil content (7.2% in 2018 compared with 5.1% in 2017). In fact, a negative correlation was observed between these two parameters in this study and previous studies on quinoa (47) but also in oilseeds such as soybean (62, 63). This is an important aspect to be considered when aiming at improving seed quality and has driven big efforts in soybean breeding (64, 65). A different explanation could be that the lipid biosynthesis-related enzymes are affected by high temperatures, decreasing oil synthesis (35). Interestingly, elevated temperatures not only affected total oil content but also the oil composition, as reported previously for oilseeds (66). Considering that most parts of the PUFA in the seeds are formed by desaturation of oleic and linoleic acids catalyzed by desaturases (37), which are enzymes that introduce double bonds (unsaturation) in the fatty acyl chains of lipids (36), one can hypothesize that those enzymes can be targets of heat stress (37, 67). This would explain the compositional changes observed between years (2017 and 2018) as well as the negative correlation found between oleic and linoleic acids specifically and between MUFA and PUFA contents, in general (Figure 8). Furthermore, fatty acid trafficking through various organelles, which is indispensable for the synthesis of C18 unsaturated fatty acids, might be affected by abiotic stressors (35, 55). This, together with the multiple associated roles that C:18 unsaturated fatty acids play under abiotic and biotic stresses, could lead to the changes observed in the oil composition (55).

In oilseed crops, such as sunflower (68, 69), rapeseed (70), or soybean (17, 37), it was shown that the fatty acid composition depends on the genotype and is determined by the temperature conditions during the seed-filling stage, both factors regulating the ratio oleic acid:linoleic acid (71). Thus, higher temperatures at the seed-filling stage resulted in larger contents of palmitic and oleic acids, while reducing linoleic levels in sunflower (71) and rapeseed (70). Also, in soybean, it was observed that elevated temperatures at the seed-filling stage cause an elevated oleic acid content and a decrease of linoleic and linolenic acids (17). On the contrary, in camelina seeds, the lipid profile was temperature-insensitive (66). In this study, the ratio of oleic acid:linoneic acid was significantly lower under high temperatures (0.32, in 2017 compared with 0.37 in 2018) contrary to what was observed in sunflower, rapeseed, or soybean. Furthermore, in olive, heat stress diminishes oleic acid content and increases linoleic and palmitic acid contents, supporting our results (72). This might be explained, at least partially, by the effect on the expression of oil biosynthetic-related genes (73).

In contrast, the major fatty acid found in quinoa seeds, the linoleic acid (which appeared in levels similar to those previously reported studies (3, 4, 12), showed larger contents in 2017 (61.0%) compared with 2018 values (58.4%) in all the quinoa cultivars tested except for Jessie, the cultivar with the shortest life cycle (in which no changes were observed). This effect could be associated with the elevated temperatures recorded in 2017 at seed filling and would explain why Jessie, affected by lower temperatures at this stage, did not show changes in the linoleic acid level. This temperature-dependent response is similar to the response observed by others (40), which showed that most parts of the quinoa cultivars analyzed yielded less PUFA contents under higher temperatures (except for the linoleic acid, whose variation was genotype-dependent). Nonetheless, it should be noted that, in this previous study, the variations of both temperature and radiation were less pronounced and, also, the sowing date changed, which, consequently, can affect other environmental parameters.

In the case of the oleic acid, the trend was also similar to what was found by other authors (40), and its content was significantly lower under elevated temperatures (2017, 19.5% compared with 2018, 21.2%), although differences related to the genotype were only found in medium-long cycle varieties (Roja and Duquesa) and Marisma. Duquesa and Roja showed a 9.6 and 12% reduction, respectively, while in Marisma, it was 12.0%. Therefore, a G × E effect was detected in the fatty acid profile, similar to what was pointed out earlier (9) and contrary to what was shown in other studies (27), in which the oil content and composition were only dependent on the genotype but with no effect related to the environmental conditions. Noteworthy, it should be highlighted that the G × E interaction was also determinant of other PUFA levels, although the high-temperature impact was genotype-dependent.

Interestingly, the palmitic acid content increased in 2017 (9.9%) compared with 2018 (9.6%), similar to what was previously observed (9), although this increase was only observed in the medium-long life cycle cultivars, mainly in Duquesa. This fatty acid decrease, together with the lower oleic acid content and the larger linoleic acid content observed in 2017, supports the idea of an effect of elevated temperatures on desaturases at transcriptional and posttranscriptional levels (74, 75).

Despite the variations observed in fatty acid composition, the total amount of SFA did not change under elevated temperatures. However, in all varieties except for Jessie and Pasto, the MUFA content was significantly lower under elevated temperatures, while, on the contrary, the PUFA content was significantly higher. These results were opposed to those found in soybean, rapeseed, or sunflower that showed higher levels of MUFA and lower levels of PUFA under elevated temperatures (66). In line with this, it should be noted that the fact that night temperatures were similar between years could result in milder changes in the fatty acid profile.

Furthermore, the ω-6 to ω-3 ratio changed between years for some cultivars. Pasto, Roja, and Duquesa showed higher ratios in 2017, due to the linoleic acid increase (ω-6) and the lower content of α-linoleic acid. The ratio ω-6/ω-3 has been considered a key aspect when aiming at preventing different chronic diseases (76). Elevated intakes of ω-6 are associated with an increase in the incidence of inflammatory diseases, such as cardiovascular diseases, cancer, or diabetes (14). Overall, the ω-6/ω-3 ratios found in the quinoa varieties analyzed in this study was approximately 9/1, while this ratio is higher than the optimal (which is considered 4/1), which is still better than the ratios found, on average, in Western diets (15.0/1–16.7/1) (14). Moreover, and compared with other seed oils, the ω-6/ω-3 ratio in quinoa is lower than the ratio found in olive (13.4/1) or corn oil (52/1) (77) and similar to the ratio found in soybean oil (7.4/1). This result highlights the effect of temperature worsening seed quality, which is genotype-dependent (78).

Interestingly, certain fatty acids that may contribute to the lower seed quality of quinoa seeds, such as the erucic acid (C22:1), did not vary with the temperature. The erucic acid is a monounsaturated very long-chain fatty acid (C22:1) whose consumption may have negative effects on health (79). It is produced by different plant species, especially in members of the brassica family (80–82), being the major component of rapeseed oils. The unchanged erucic acid levels found in this study differ from what was found in rapeseed, in which elevated temperatures caused a reduced erucic acid content (83, 84). Nonetheless, the variability of the erucic acid contents that appeared in 2018, for most of the quinoa varieties studied, may reflect an influence of the environmental conditions and genotype in this fatty acid content that should be further analyzed.

Also, it should be highlighted that, in this study, some fatty acids, which are infrequent in plants, were found (in small amounts) in the quinoa seeds analyzed (Supplementary Figure 1). These included the pentadecanoic acid (C15:1, <0.1%), the margaric acid (C17:0, <0.1%), and the margaroleic acid (C17:1, <0.2%) (Table 1), and some of which were already identified in quinoa seeds (85).

The correlation between the parameters in this study tested revealed interesting aspects related to the fat content and mineral nutrients including the relation between fat content and seed or straw P contents, which showed a negative correlation of −0.9 and −0.9, respectively. The mineral content of quinoa seeds showed that P was higher under elevated temperatures, when comparing among varieties, in Jessi (33). This might be an interesting aspect as, based on the current correlogram analysis, the P content may indicate oil compositional changes in quinoa. This would be supported by the correlations found between P (in the seeds and in the straws) with the palmitoleic acid, oleic acid (negatively), and linoleic acid (positively) contents (Figure 8). As described in previous studies, P fertilization rates might affect oil yields in crops (86, 87), which seems to be P rate-dependent (88). Nonetheless, more experiments should be performed in quinoa to further explore this negative relation in order to optimize the fertilization rates and seed quality. Also, the seed N content might be related to the fatty acid composition of quinoa seeds. In this study, N showed strong negative correlations with the fatty acid profile, evidencing the link between this mineral and seed oil quantity (−0.9) and quality. For example, N showed a negative correlation with MUFA (−0.7), palmitoleic (C16:1, −0.9), gadoleic (20:1, −0.8), and erucic (C22:1, −0.8) acid contents and positive with the margaric (C17:0, 0.87) and linoleic (C18:2, 0.67) acids. In line with this, the N content presented higher contents under elevated temperatures (in 2017) although no genotype-dependent variations were found among the varieties analyzed in this study (6). Despite the fact that N fertilization might affect the oil content and composition of seeds (89), further studies are required to elucidate the relationship between this mineral use efficiency, the fat contents, and the environmental influence on these parameters. Furthermore, in quinoa seeds, the total fat and FB contents have been positively correlated with yield parameters such as seed weight, although this relation was genotype- and sowing date-dependent (90). In this study, seed FB showed a strong negative correlation with the CH content (−1) but also with total fat (−0.7) and erucic acid (−0.6) contents, which may reflect relations between FB and oil contents, impacting seed quality. In line with this, it should be mentioned that, among the varieties analyzed, Roja was the one showing a higher FB content (22.71%) and Pasto the one with the lowest percentage (12.6%) (6).

## Conclusion

Seed oil composition is a determinant of the nutritional quality of quinoa. Although quinoa is not an oilseed crop, the fatty acid composition is remarkable due to the oil profile rich in unsaturated fatty acids. In this study, the effect of high temperatures was evaluated under field conditions and resulted in lower oil content and detrimental effects on the fatty acid composition. These effects included a lower ratio of oleic acid:linoleic acid and larger ω-6 to ω-3 ratios. Overall, the results presented in this study highlight the environmental control of seed quality-related parameters such as the oil content and composition, and how these are also genotype and G × E dependent. Furthermore, the correlations found between mineral nutrients such as P and the oleic and linoleic acids emphasize the possibility of optimizing the fertilization to improve oil quality in quinoa.

## Data Availability Statement

The original contributions presented in the study are included in the article/Supplementary Material, further inquiries can be directed to the corresponding author.

## Author Contributions

JM, MR, and MJR conceived of the presented idea. JM and MR designed the experiment, researched and analyzed the background literature, wrote the manuscript, and including interpretations. MJR and PC researched and analyzed the background literature, carried out the chemical analysis, and wrote portions of the manuscript, and including interpretations. SG-R performed the statistical analysis and revised the manuscript critically for intellectual content. JM and VC carried out the field experiments and processed the data. All authors contributed to this study and approved the submitted version.

## Conflict of Interest

The authors declare that the research was conducted in the absence of any commercial or financial relationships that could be construed as a potential conflict of interest.

## Publisher’s Note

All claims expressed in this article are solely those of the authors and do not necessarily represent those of their affiliated organizations, or those of the publisher, the editors and the reviewers. Any product that may be evaluated in this article, or claim that may be made by its manufacturer, is not guaranteed or endorsed by the publisher.

## Supplementary Material

Supplementary Figure 1 Quinoa seed samples were analyzed by gas chromatography-mass spectrometry (GC-MS) to confirm the identification of the FAMEs. **(A,D)** Chromatogram of C15:0 and C17:1 fatty acids identified by gas chromatography/mass (GC/MS) for a representative quinoa sample (red line) and for the FAME standard (green line). **(B,E)** Mass spectrum of C15:0 and C17:1 peaks in full scan mode and **(C,F)** the NIST spectral library for C15:0 and C17:1. Images were obtained at *Servicios de Apoyo a la Investigación* (SAIUEX), Universidad de Extremadura, Spain. The identification of the sample peaks [**(A)**: C15:0; **(D)**: C17:1] was conducted by comparing their retention times with those of a commercial standard mixture of FAME. The peaks were confirmed by mass spectrometry comparting their mass spectrum [**(B)**: C15:0; **(E)**: C17:1] with the NIST library [**(C)**: C15:0; **(F)**: C17:1] based on the specific fragmentation and the charge/mass ratio.
