# Supplementary figures and images for: Changes in Quinoa Seed Fatty Acid Profile Under Heat Stress Field Conditions
Source: Front Nutr. 2022 Mar 28;9:820010. doi: 10.3389/fnut.2022.820010 (PMC8996139; doi:10.3389/fnut.2022.820010)

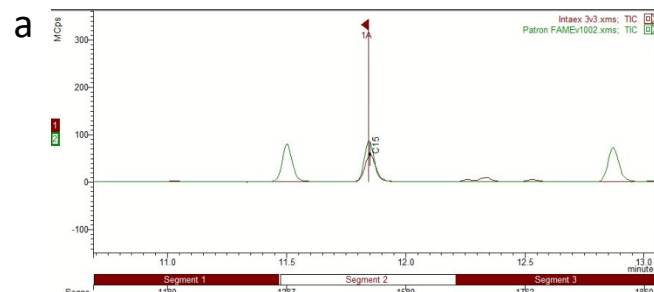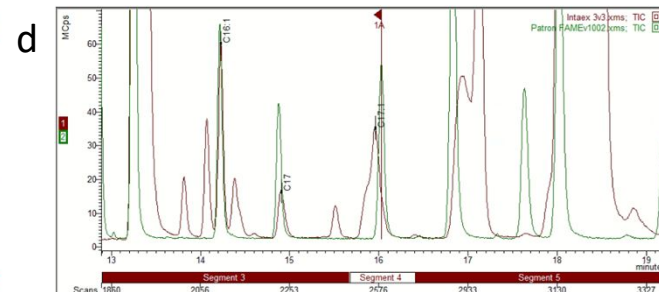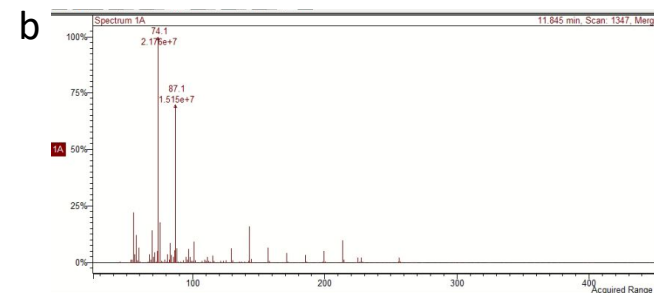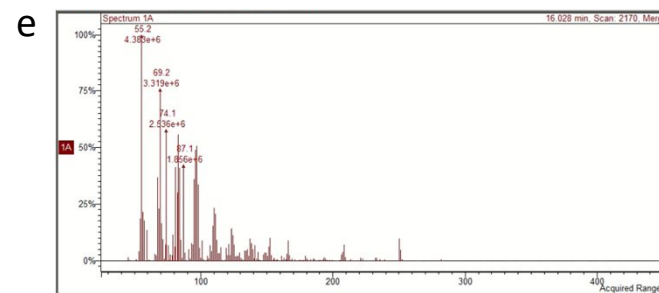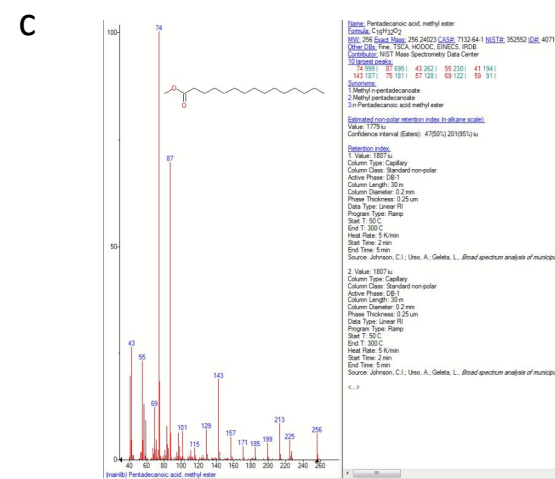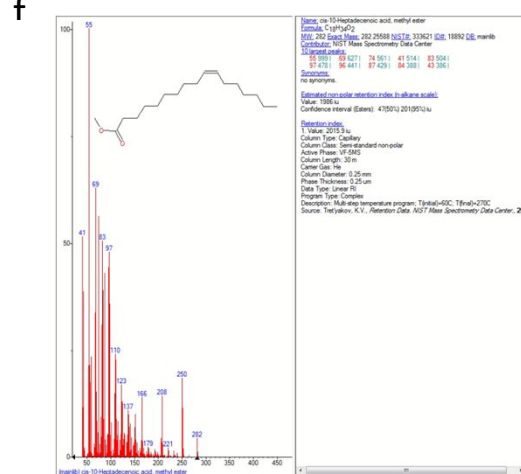

Supplement: Supplementary Figure 1 — Quinoa seed samples were analyzed by gas chromatography-mass spectrometry (GC-MS) to confirm the identification of the FAMEs. (A,D) Chromatogram of C15:0 and C17:1 fatty acids identified by gas chromatography/mass (GC/MS) for a representative quinoa sample (red line) and for the FAME standard (green line). (B,E) Mass spectrum of C15:0 and C17:1 peaks in full scan mode and (C,F) the NIST spectral library for C15:0 and C17:1. Images were obtained at Servicios de Apoyo a la Investigación (SAIUEX), Universidad de Extremadura, Spain. The identification of the sample peaks [(A): C15:0; (D): C17:1] was conducted by comparing their retention times with those of a commercial standard mixture of FAME. The peaks were confirmed by mass spectrometry comparting their mass spectrum [(B): C15:0; (E): C17:1] with the NIST library [(C): C15:0; (F): C17:1] based on the specific fragmentation and the charge/mass ratio. [file Image_1.pdf]
